# Developing a theory-based instrument to assess the impact of continuing professional development activities on clinical practice: a study protocol

**DOI:** 10.1186/1748-5908-6-17

**Published:** 2011-03-07

**Authors:** France Légaré, Francine Borduas, André Jacques, Réjean Laprise, Gilles Voyer, Andrée Boucher, Francesca Luconi, Michel Rousseau, Michel Labrecque, Joan Sargeant, Jeremy Grimshaw, Gaston Godin

**Affiliations:** 1 Research Center of the Centre Hospitalier Universitaire de Québec, Hospital St-François D'Assise, Québec, Canada; 2 Continuing Professional Development Office, Faculty of Medicine, Université Laval, Québec, Canada; 3 Practice Enhancement Division, Collège des médecins du Québec, Montréal, Canada; 4 Office of Professional Development, Fédération des médecins spécialistes du Québec, Montréal, Canada; 5 Faculty of Medicine and Health Sciences, Université de Sherbrooke, Sherbrooke, Canada; 6 Faculty of Medicine, Université de Montréal, Montréal, Canada; 7 Center for Continuing Health Professional Education, Faculty of Medicine, McGill University, Montréal, Canada; 8 Departement of Family Medicine and Emergency Medicine, Université Laval, Québec, Canada; 9 Division of Medical Education, Faculty of Medicine, Dalhousie University, Halifax, Canada; 10 Clinical Epidemiology Program, Ottawa Hospital Research Institute, Ottawa, Canada; 11 Faculty of Nursing, Université Laval, Québec, Canada

## Abstract

**Background:**

Continuing professional development (CPD) is one of the principal means by which health professionals (i.e. primary care physicians and specialists) maintain, improve, and broaden the knowledge and skills required for optimal patient care and safety. However, the lack of a widely accepted instrument to assess the impact of CPD activities on clinical practice thwarts researchers' comparisons of the effectiveness of CPD activities. Using an integrated model for the study of healthcare professionals' behaviour, our objective is to develop a theory-based, valid, reliable global instrument to assess the impact of accredited CPD activities on clinical practice.

**Methods:**

Phase 1: We will analyze the instruments identified in a systematic review of factors influencing health professionals' behaviours using criteria that reflect the literature on measurement development and CPD decision makers' priorities. The outcome of this phase will be an inventory of instruments based on social cognitive theories. Phase 2: Working from this inventory, the most relevant instruments and their related items for assessing the concepts listed in the integrated model will be selected. Through an e-Delphi process, we will verify whether these instruments are acceptable, what aspects need revision, and whether important items are missing and should be added. The outcome of this phase will be a new global instrument integrating the most relevant tools to fit our integrated model of healthcare professionals' behaviour. Phase 3: Two data collections are planned: (1) a test-retest of the new instrument, including item analysis, to assess its reliability and (2) a study using the instrument before and after CPD activities with a randomly selected control group to explore the instrument's mere-measurement effect. Phase 4: We will conduct individual interviews and focus groups with key stakeholders to identify anticipated barriers and enablers for implementing the new instrument in CPD practice. Phase 5: Drawing on the results from the previous phases, we will use consensus-building methods to develop with the decision makers a plan to implement the new instrument.

**Discussion:**

This project proposes to give stakeholders a theory-based global instrument to validly and reliably measure the impacts of CPD activities on clinical practice, thus laying the groundwork for more targeted and effective knowledge-translation interventions in the future.

## Background

In Canada, it is assumed that continuing professional development (CPD), which encompasses continuing medical education (CME) [1], plays an important role in maintaining and improving the quality and efficiency of the healthcare system [2] by translating evidence into clinical practice [3]. In other words, CPD serves as an important knowledge-translation strategy and is one potential approach that could be incorporated into the Knowledge to Action Process (KTA) framework [4]. The KTA framework, which explains how knowledge is produced and implemented in healthcare, contains two parts: the *knowledge creation cycle *and the *action cycle*. While the first cycle comprises the process of creating knowledge, the second one constitutes the process of applying the knowledge thus created. By translating knowledge and evidence into practice, CPD pertains to the action cycle [5]. Designed to improve performance in healthcare practices and, ultimately, health outcomes, CPD strategies follow the dynamic and iterative process for knowledge translation.

As in other educational disciplines, most evaluation frameworks used in CPD are derived from Kirkpatrick's model [6]. This model assesses training effectiveness by measuring participants' reactions to an educational activity (level 1); changes in participants' knowledge, skills, or attitudes (level 2); transfer of learning to practice/observed changes in behaviour (level 3); and finally, the results of the newly acquired behaviour on organizational outcomes such as productivity and quality (level 4). According to this model, the effects of current approaches to the assessment of the impact of accredited CPD activities should ideally be evaluated focusing on participants' participation, satisfaction, and changes in knowledge, behaviour, and patient outcomes [7], [8], [9]. In practice, however, most CPD providers only assess levels 1 and 2 outcomes using pre- and post-activity self-administered questionnaires. Although the impacts of levels 3 and 4 have been measured in the context of research projects using health services methods [10], [11], CPD providers are still struggling to find reliable ways to measure these impacts on a routine basis.

Godin and colleagues (2008) proposed an integrated conceptual model to predict behaviour change in healthcare professionals that offers a clear basis for developing a valid and reliable measurement instrument to assess CPD impacts on clinical practice (Kirkpatrick's level 3 outcomes). Since individual decisions are often central to the adoption of clinically related behaviours, theories providing information about cognitive mechanisms underlying behaviours help provide direction to behaviour-change interventions targeting healthcare professionals. The results of a systematic review of 78 studies suggested that a number of constructs originating from social cognitive theories were the most promising for assessing behaviour change in health professionals [12].

Our study aims to: (a) meet CPD providers' needs for high-quality evaluation methods that are based on well-established theories for predicting clinical behaviour and intentions to change of healthcare professionals (level 3 of Kirkpatrick's model) and (b) establish guidelines for the reliability and validity of those methods [13]. The integrated model proposed by Godin and colleagues (2008) has identified key constructs likely related to behaviour, and we now want to develop an instrument that assesses the constructs in this framework as a proxy for predicting changes in behaviour in CPD activities.

### Study team

Composed of knowledge-translation researchers and CPD decision makers, the partnership responsible for this study protocol has been in effect since 2006 and organized a successful meeting in February 2009. It now proposes to act as a model of the interaction encouraged by the Canadian Institute of Health Research's Institute of Health Services and Policy Research (IHSPR) and shape how CPD activities will be validly and reliably evaluated by the members of the Québec Council for Medical CPD in coming years. This project was born out of the needs of CPD educators and decision makers and has evolved over a three-year period in which a sound foundation for a collaborative research project has been laid.

## Methods

This research and development project will be carried out in five phases.

### Phase 1: identification and critical appraisal of existing instruments

Of the 78 studies included in a systematic review of studies of factors influencing health professionals' behaviour, we identified 49 used tools with acceptable psychometric qualities [12]. These studies will be retrieved for details about the instruments in question. Several methods will be used to ensure the completeness of the information regarding each instrument. If the paper does not provide detailed information, the authors will be contacted. The expected outcome of this phase is an inventory of instruments based on social cognitive theories. This initial inventory will be validated and enriched by updating the literature search and contacting experts in the field (CPD specialists, health services researchers, and social psychologists) through diverse mailing lists.

The instruments thus identified will be analyzed using criteria based on the literature on measurement development (*e.g.*, instructional design principles requirement, psychometric properties) [14], [15], [16]. They will also be analyzed based on criteria that CPD decision makers deem important (*e.g.*, be able to be used by a large number of physicians in different clinical areas and contexts and be inexpensive to administer). To fulfill these tasks, two research assistants will independently extract data using a standardized form that will be revised at the beginning of the research project by team members to ensure that it fits decision makers' needs.

### Phase 2: assessment of the instrument's applicability--development of a new global instrument based on social cognitive theories

The goal of phase 2 is to assess the applicability of and adapt any instruments identified in phase 1. We will create a seven-person Development Committee comprised of researchers, CPD decision makers, and an external expert from each of the CPD and knowledge-translation research communities. Members of this committee will largely be from the research team. Examining each instrument selected in phase 1, the Development Committee will choose the most relevant instruments and their related items for assessing the variables (concepts) listed in our integrated conceptual model. Once this task is complete and all items are integrated into a new global instrument, an e-Delphi process will be used to check its face validity and likely acceptability and utility in CPD settings. Acceptability will be assessed with criteria used in phase 1. The resulting global instrument will also be examined for (a) its appropriateness or relevance to behaviours expected to occur after CPD activities, (b) its grammar and readability, and (c) the appearance of bias in two rounds of planned contacts within the entire research team and its network.

Once the team and its networks have provided their feedback, the Development Committee will review the final list of selected instruments and their respective items grouped around variables (concepts) of the integrated conceptual model and ensure that the new global instrument (*i.e.*, one that includes selected instruments) continues to meet the criteria elaborated above. The Development Committee may revise or discard items and variables as necessary. We will begin by developing a French version of the global instrument.

### Phase 3: assessment of the reliability and validity of the new theory-based global instrument

The objective of phase 3 is to assess the reliability and validity of the new theory-based global instrument, including its ability to predict intentions and behaviour, and also its sensitivity to change in response to CPD activities in a group of physicians who have participated in an accredited CPD activity.

### Study population and recruitment strategy

Participants will be recruited from attendees of the CPD activities offered by the CPD decision makers and collaborators to this project. Each year, thousands of educational activities are offered through this network. These institutions and organisations reach the vast majority of the 16,000 practising physicians in the province of Québec. Eligibility criteria for CPD activities will include the following: (a) accreditation by one of the CPD decision makers and collaborators to this project; (b) group-based, live activities carried out with groups of 50 participants or less; (c) a focus on behaviour change for any topic or content as stated in the learning objectives established for the activity; (d) occurrence in any setting (*e.g.*, university, conference venue, practice setting); (e) use of any or a combination of instructional methods (*e.g.*, didactic lectures, workshops, case studies, demonstrations) and material (*e.g.*, audience response systems, videos, card games, real or simulated patients), which must include at least 25% of interactivity; (f) conduct as a one-time intervention; and (g) duration of between at least one and three hours. Individual activities embedded within large programs offering several activities at one setting, such as a two-day conference, will be eligible.

Eligibility criteria for physicians will include the following: (a) attending an eligible live CPD activity, (b) being active in clinical practice for a six-month period following the indexed CPD activity, (c) being accessible for a phone interview three months after the indexed CPD activity, (d) speaking fluent French, and (e) not having participated in this study previously.

A research assistant will attend a number of eligible CPD activities chosen at random from one of the CPD decision-makers' and collaborators' networks. At registration in the CPD activity, the assistant will enroll participants and ask them to complete a consent form and a sociodemographic questionnaire. The research assistant will also collect data on the CPD activity to which the participants are exposed.

### Data collected

Participant-level data collected for the study will include the participants' sociodemographic details (*e.g.*, gender, age, type of medical speciality, and years of experience in current practice). CPD activity-level data collected for the study will include CPD activity attended, the clinical area of the activity, the type of needs assessment conducted, the theoretical foundation, the length, the number and type of instructional methods used (*e.g.*, group discussion, role-play, video, touch pad) [17], the number of faculties involved in its design and presentation, and the source of funding. In addition, the content of the CPD activity relating to basic evidence-based information deemed necessary for making quality medical decisions will also be assessed.

In accordance with the integrated conceptual model proposed by Godin and colleagues (2008) to predict behaviour of healthcare professionals, our new global instrument will basically assess participants' information, namely, beliefs about consequences and capabilities, social influences, moral norms, perceptions of role and identity, and their intentions, habits, and past behaviours.

### Data collection procedures

Two data collections are planned: (1) a test-retest of the new instrument for assessing its reliability and item analysis and (2) a before-and-after study to test the validity of the instrument.

First, a group of 50 participating physicians (25 family physicians and 25 specialists) who attended an eligible CPD activity will be asked to complete the instrument immediately after the CPD activity and to use the same instrument again after two weeks. The research assistant will offer to mail the instrument to the physicians. This first data collection will provide the data for assessing both intra-subject reliability at two weeks and the internal reliability of the instrument. Reliability of the scales will be assessed with Cronbach's alpha for internal consistency and with an intraclass correlation coefficient for stability. It will thus be possible to modify the instrument based on this first set of results. The final choice of the set of items will be based on their indices of distribution and discrimination and their theoretical importance.

Second, we will use a before-and-after study design. In eligible CPD activities, half of the participants, randomly selected, will be asked to complete the evaluation instrument both before and at the end of the CPD activity, and half of the participants will be asked to complete the evaluation instrument after the CPD activity. Data from the participants who will complete the evaluation instrument before and after the CPD activity will allow us to verify the instrument's capacity to detect differences in constructs of the integrated model. Comparing results from participants who completed the instrument before and after the CPD activity and those who completed it only after the activity will allow us to explore the mere-measurement effect (*i.e.*, if everyone completes the questionnaire twice, it is not possible to ascertain whether the effect can be attributed to the CPD activity or to the action of completing the questionnaire) [18].

All participants will be contacted three months later by phone in order to collect self-reported clinical behaviour, as targeted in the specific CPD activity. These data will be used to estimate the instrument's effectiveness in predicting behaviour. In accordance with the results of the systematic review of factors predicting behaviour in healthcare professionals [12], self-reported behaviour will be used as a proxy. Although more robust approaches to measure behaviour, such as chart audit or standardized simulated patients, would be preferable, these methods are not feasible in the context of this project.

### Sample size

For the before-and-after data collection, we will seek the participation of 500 CPD enrollees: 125 family physicians/before and after, 125 family physicians/after only, 125 specialists/before and after, and 125 specialists/after only. Apart from sociodemographic results, the integrated model currently proposes seven essential concepts to predict behaviour in healthcare professionals [12]. Each concept represents a construct. Usually, each construct is assessed with 3 to a maximum of 10 items. A minimum of 100 data entries are needed for an exploratory factor analysis (EFA) [19]. Also, a minimum of 10 data entries are needed per parameter to make estimations for confirmatory factor analysis (CFA) [19]. For example, for a construct that is assessed with 10 items, we will have a minimum of 33 parameters to estimate. Consequently, 430 participants should provide an adequate sample size for performing both EFA and CFA for the two groups together (family physicians and specialists), as well as some of the other planned validity and reliability analyses. We will need 473 physicians to consider a 10% of potential losses to follow-up. Therefore, the recruitment of 500 physicians (250 family physicians and 250 specialists) will provide an adequate power for the analysis.

### Statistical analyses

Using EFA, we will explore the factorial structure within the potential seven constructs describing essential concepts to predict behaviour of healthcare professionals. We will then use CFA to assess construct validity.

We will also assess the relationships between the constructs based on the proposed integrated model using path analysis. Observation of the expected relationships will be further proof of the validity of the model and its constructs. The difference between family physicians and specialists for each construct will be assessed with a multiple-group analysis. Also, in participants who completed the instrument before and after the CPD activity, the effect of participating in a CPD activity and change in the physician's intentions with a paired student *t*-test will be verified. Our *a priori *hypothesis is that physician's intentions will be positively associated with participation in the CPD activity. Physician's intentions after the CPD activity calculated in this group will be contrasted with results obtained in the control group to explore the mere-measurement effect [18]. Predictive validity will be estimated by correlating the developed measurement instrument score and ratings from participants on the intention construct with self-reported behaviour at three months. Our *a priori *hypothesis is that the measurement instrument will correlate in the expected direction with self-reported behaviour as well as with changes in self-reported behaviour.

### Phase 4: assessment of the acceptability of the new instrument by decision makers and CPD participants

The objective of phase 4 is to explore the acceptability of the new global instrument by CPD decision makers and gather suggestions of factors that might influence its implementation. Data collection will consist of individual interviews and focus groups with key stakeholders. We will use the contact network of team members to purposefully select participants from within four groups: (1) Québec's key stakeholder CPD organizations, (2) primary care physicians, (3) specialists, and (4) representatives of Québec's Ministry of Health and Social Services. The aim is to highlight anticipated barriers and enablers for implementing the new instrument in CPD practices.

We will collect the participants' sociodemographic characteristics and additional information on their organizations. Interviews and focus groups will be structured according to an interview guide that will facilitate semi-structured discussions regarding the instrument. Examples of questions to be included in the interview guide are as follows: Who do you think might have a favourable/negative attitude to this measurement instrument? Can you think of barriers and enablers for implementing this instrument in CPD practice?

All interviews and focus groups will be tape-recorded and transcribed verbatim. Analysis of the transcripts will occur concurrently with data collection in both the focus groups and the individual interviews and will use a constant comparative method. Briefly, units of data will be categorized and new units compared with previously identified ones in order to develop or saturate each category [20]. NVivo (QSR International, Cambridge, MA, USA) software for qualitative analyses will be used to support data collection, organization, and analysis.

Internal validity of the study will include member checking [21]. A summary of the interpretations of the interviews and the focus groups will be sent to each participant, who will be invited to make comments and corrections. Subsequently, a summary of the barriers and enablers for implementing the measurement instrument in CPD practice and proposed actions to be taken will be circulated and discussed in a conference call with the research team. A report of the results from phase 4 activity will be produced to be used in phase 5.

### Phase 5: development of a consensus among CPD decision makers regarding an implementation plan for the new instrument

This research activity will involve meeting with Québec's medical CPD consultative coordinating body. The research methods used in this phase will be based on consensus-building methods, which will include convening, clarifying responsibilities, deliberating, deciding, and implementing agreement. Consensus building is defined as the process of seeking unanimous agreement [22]. The research team will draw on the results from the above-mentioned research activities: (a) the new measurement instrument and its characteristics (*e.g.*, items, validity, and reliability data); (b) a summary of the barriers and enablers as perceived by key stakeholders for implementing this instrument in CPD practice; and (c) proposed actions to assess the impact of CPD activities. Details of the results of these research activities will be sent to team members in preparation for a face-to-face meeting. The face-to-face meeting will be structured around a specific goal: building consensus regarding an implementation plan for the new instrument to assess the impact of CPD activities. The meeting will be led by an external facilitator. The agenda will aim at achieving agreement on a final plan for implementing the new instrument.

Once the instrument has been found to be valid and reliable, we will create an English version using translation and back-translation methods [23].

## Discussion

This project will serve not only to develop an appropriate instrument to assess the impact of accredited CPD activities on physicians' performance but also to test the integrated conceptual model for the study of healthcare professionals' behaviours and intentions proposed by Godin and colleagues (2008). We acknowledge that external factors such as workplace characteristics, financial incentives, and patient expectations may influence the results of an accredited CPD activity on physicians' performance. However, the principal target of CPD interventions is not organizational or environmental change but individual change. For that reason, our instrument will be designed to measure the impacts of CPD on behavioural change at the individual level. "Individuals are essential units of health education and health behaviour theory, research, and practice. This does not mean that the individual is the only or necessarily the most important unit of intervention. But all other units, whether they are groups, organizations, worksites, communities, or larger units, are composed of individuals." [24] A reliable assessment instrument based on an integrated conceptual model that links cognition and behaviour change has the potential to be acceptable to CPD decision makers and participants across the province of Québec. In addition, such an instrument could provide an exceptional opportunity for monitoring and adapting CPD interventions so that they can contribute more effectively to improving healthcare practices and, by extension, the quality of the healthcare system as a whole.

## Competing interests

The authors declare that they have no competing interests.

## Authors' contributions

All authors collectively drafted the study protocol and approved the final manuscript. FL is its guarantor.
